# Diverse Bacterial Communities From Qaidam Basin of the Qinghai–Tibet Plateau: Insights Into Variations in Bacterial Diversity Across Different Regions

**DOI:** 10.3389/fmicb.2020.554105

**Published:** 2020-09-18

**Authors:** Wei Zhang, Ali Bahadur, Gaosen Zhang, Binglin Zhang, Xiukun Wu, Tuo Chen, Guangxiu Liu

**Affiliations:** ^1^Key Laboratory of Desert and Desertification, Northwest Institute of Eco-Environment and Resources, Chinese Academy of Sciences, Lanzhou, China; ^2^Key Laboratory of Extreme Environmental Microbial Resources and Engineering, Lanzhou, China; ^3^State Key Laboratory of Cryospheric Sciences, Northwest Institute of Eco-Environment and Resources, Chinese Academy of Sciences, Lanzhou, China

**Keywords:** community dynamics, soil depth profiles, desert ecosystem, driving factors, Qaidam Basin

## Abstract

The Qaidam Basin of the Qinghai–Tibet Plateau is a cold, hyper-arid desert that presents extreme challenges to microbial communities. As little is known about variations between surface and subsurface microbial communities, high-throughput DNA sequencing was used in this study to profile bacterial communities of the soil samples collected at different depths in three regions in the Qaidam Basin. The α-diversity indices (Chao, Shannon, and Simpson) indicated that bacterial abundance and diversity were higher in the east and the high-elevation regions compared to the west region. In general, *Firmicutes* was dominant in the west region, while *Proteobacteria* and *Acidobacteria* were dominant in the east and the high-elevation regions. The structure of the bacterial communities differed greatly across regions, being strongly correlated with total organic carbon (TOC) and total nitrogen (TN) content. The differences in bacterial communities between the surface and the subsurface soil samples were smaller than the differences across the regions. Network analyses of environmental factors and bacterial genera indicated significant positive correlations in all regions. Overall, our study provides evidence that TOC and TN are the best predictors of both surface and subsurface bacterial communities across the Qaidam Basin. This study concludes that the bacterial community structure is influenced by both the spatial distance and the local environment, but environmental factors are the primary drivers of bacterial spatial patterns in the Qaidam Basin.

## Introduction

Cold desert ecosystems provide ideal model systems for investigating soil microbial diversity, as the microbial communities play vital roles in desert ecosystems, including the regulation of the biogeochemical cycles of key nutrients (Torsvik et al., 2002). Unraveling the factors that shape microbial community diversity is crucial for predicting ecosystem responses to environmental changes in order to improve variation in various ecosystems (Chase and Myers, 2011; Sajjad et al., 2018). With the current advances in metagenomic technologies (Zhou et al., 2015), the soil microbial community distribution and diversity have been intensively studied in a wide variety of environments (Fierer and Jackson, 2006; Thompson et al., 2017), including in harsh desert soil. These studies have suggested that environmental factors greatly shape the soil microbial community structure in cold desert ecosystems.

Studies in cold desert regions have focused entirely on bacterial diversity and communities in salt lakes, reporting on halophilic bacteria and bacterial activity associated with gas and water (Wang et al., 2014; Chen et al., 2015; Han et al., 2017; Shen, 2017; Stovicek et al., 2017). Soil bacterial communities may influence nutrient cycles and availability and maintain soil quality. Mounting evidence supports the roles of driving factors such as total nitrogen (TN), total organic carbon (TOC), pH, and elevation in structuring bacterial communities (Maestre et al., 2015; Prober et al., 2015). In particular, multiple desert studies have shown that moisture is a critical driving factor influencing bacterial community structures, assembly, and colonization (Stomeo et al., 2012; Valverde et al., 2015). The bacterial community represents a major part of the desert soil biodiversity, and the bacteria are involved in controlling the fluctuations in soil physicochemical properties (Makhalanyane et al., 2015). Moreover, being a low-temperature ecosystem, the cold desert might inhabit potential ancient pathogens and/or antibiotic-resistant genes that could unlock due to global warming (Sajjad et al., 2020b). Therefore, it is important to study cold desert soil bacterial communities and their driving factors.

The bacterial community distribution in surface desert soil has been well characterized in many studies. According to Fierer et al. (2003), soil microorganisms are collectively the most active microorganisms and have the densest populations. The subsurface soil also represents one of the most diverse ecosystems, with a community of interacting microorganisms that have important roles in soil formation, groundwater maintenance, ecosystem biogeochemical processes such as nutrient cycling, and maintenance of soil respiration (Hiebert and Bennett, 1992; Richter and Markewitz, 1995; Fierer et al., 2003; Wagg et al., 2014; Zhou et al., 2014). Given that the subsurface soil environments are different from the surface soil environments (Fierer et al., 2003; Li et al., 2014), the subsurface soil might contain different microbial communities (Fierer et al., 2003; Chu et al., 2016). Despite recent advances in our understanding of the soil bacterial communities in hyper-arid regions (Van Horn et al., 2013), the geographical distribution of subsurface soil bacterial communities remains poorly understood. Previous studies on the bacterial composition of surface and subsurface soils have focused on single locations (Goberna et al., 2006; Xiang et al., 2008; Serkebaeva et al., 2013; Li et al., 2014). The bacterial community compositions differed greatly by soil depth, and soil properties have been reported to be the driving factors responsible for the significant differences in bacterial community compositions (Fierer et al., 2003; LaMontagne et al., 2003; Chu et al., 2016). Current knowledge regarding how and to what extent physiochemical driving factors influence the bacterial diversity in cold desert soil layers across different regions is very limited.

An in-depth exploration of soil bacterial diversity in the cold desert habitats of the Qaidam Basin region will not only improve our knowledge of bacterial ecology under extreme stresses but also provide insights into the potential effects of environmental changes in this endangered region (Xing et al., 2019). The Qaidam Basin is the main high-altitude cold desert in China, and it is thermally unstable and is ecologically sensitive to climate change (Dai, 2012). The environmental stresses are highly pronounced in this region; in particular, due to the extremely cold environment, soil nutrient availability is limited (Han et al., 2017; Xing et al., 2019). The west region is higher than the east region, and the high-elevation region tends to be higher still and has fewer passes through which the wind can travel from west to east (Li et al., 2016). The low anthropogenic activity and extreme soil conditions may give rise to soil bacterial communities that are distinct from those in other desert regions. The largely low nutrient availability, different wind direction, and lack of disturbance by humans make these regions an ideal setting to study the natural distributions of soil bacteria. The distributions of bacterial communities in this unique cold desert soil remain largely unexplored, and the variation in the structures of bacterial communities and their associations with the soil properties are not clear. Therefore, we believe that the Qaidam Basin provides an interesting ecosystem (with taxonomically diverse bacteria) to explore.

To this end, here we conducted comprehensive analyses of bacterial diversity and the factors influencing the diversity in the Qaidam Basin of the Qinghai–Tibet Plateau. We collected surface (0–10 cm) and subsurface (50 cm) soil samples at appropriate locations across the Qaidam Basin region spots. Based on the bacterial community structure clustering, the samples were categorized into three groups: east (E), west (W), and high-elevation (H) regions. These regions may have distinct soil bacterial communities compared to adjacent regions in the Qinghai–Tibet Plateau due to the harsh soil conditions of the Qaidam Basin. To investigate the influence of environmental factors on soil bacterial diversity and community structure within and across the different regions, we performed bacterial community analysis using 16S rRNA gene sequencing for each soil sample. In addition, we investigated the environmental factors that are likely to influence the diversity and the composition of soil bacterial communities. The work presented here sheds light on the soil bacterial ecology in the Qaidam Basin of the Qinghai–Tibet Plateau.

## Materials and Methods

### Site Location Description and Soil Sampling

**FIGURE 1:**
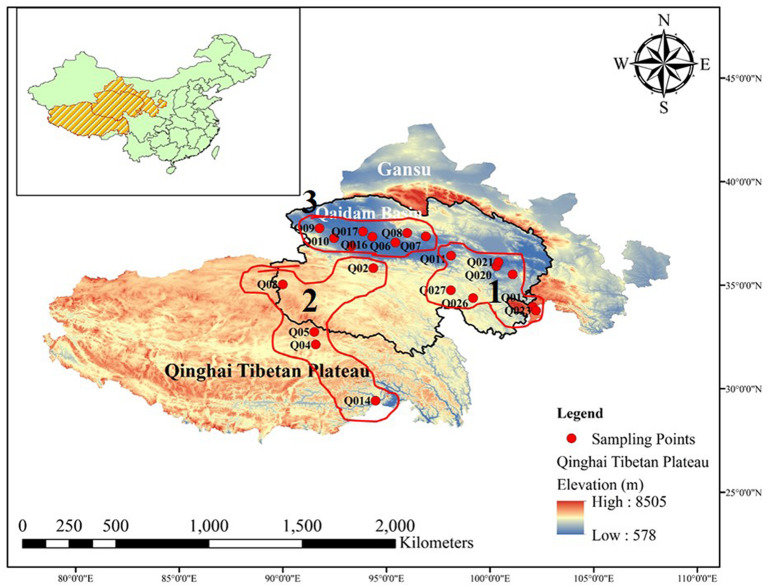
Map of Qinghai Tibetan Plateau, China. Geographical distribution of the 54 sites where bacterial diversity samples and environmental data were collected. All the sampled sites are marked by red circles. The panel insert highlights the location of the Qaidam Basin in the Qinghai Tibetan Plateau of China.

The Qaidam Basin is located on the northern edge of the Qinghai–Tibet Plateau in China (Figure 1). This region has an average elevation of around 2,700 m.a.s.l., while the surrounding mountains reach elevations of >5,000 m.a.s.l. The region investigated in this study included both the Qaidam Basin interior and the surrounding sites, located between longitudes 91–100°E and latitudes 35–37°N. The total area is about 2,60,000 km^2^. The mean annual temperature is <5°C, and the mean annual precipitation ranges from 100 mm in the southeast to <20 mm in the northwest. The potential mean annual evaporation can be 100 times higher than the precipitation.

We collected soil samples across the desert region of the Qaidam Basin interior and the surrounding area. Based on the bacterial community structure clustering, the samples were categorized into three groups: E, W, and H regions. We collected both surface soil at a depth of 0–10 cm (E0, W0, and H0) and subsurface soil at a depth of 50 cm (E5, W5, and H5). The three regions were comparatively non-uniformly distributed across the Qaidam Basin. On May 15, 2017, we collected nine soil sample replicates from each of the three regions (27 samples) at each of the two soil depths (0–10 and 50 cm), leading to 54 samples in total. Within each of the nine replicates, nine soil cores were taken and pooled to create a composite soil sample. All soil samples were sealed in sterile polyethylene bags, refrigerated, and then taken back to the laboratory as quickly as possible. The soil samples were then sieved through a 2-mm mesh to eliminate residues, and then each homogenized soil sample was divided into two parts: one was air-dried and kept at 4°C to determine the soil properties, while the other was stored at −80°C prior to DNA extraction for bacterial community profiling.

### Analyses of Soil Properties

To investigate the environmental factors that might govern the spatial distribution of soil bacteria, soil properties were analyzed, comprising soil pH, water content (WC), TOC, and TN. Soil pH was measured using a pH meter (PT-10; Sartorius, Göttingen, Germany), with a fresh-soil-to-water ratio of 1:2.5. Soil WC was calculated gravimetrically by drying the soil to a constant weight at 105°C. TOC and TN were determined using an elemental analyzer (Vario EL cube; Germany) (Liebner et al., 2009). In addition, daily meteorological data were downloaded from the China Meteorological Data Service Center website^1^. The data used in the analysis represented the average over 30 years (1981–2010) (Supplementary Figure 1).

### DNA Extraction and PCR Amplification

Total genomic DNA was extracted from 0.5 g of each frozen soil sample using a Power Soil DNA kit (Qiagen, Hilden, Germany) according to the manufacturer’s instructions. The bacterial 16S rRNA genes (V3 and V4 regions) were polymerase chain reaction (PC)-amplified using the universal primers 338F and 806R with barcode and adapter sequences. The PCR amplification was performed using 5× FastPfu Buffer (4 μl), 2.5 mM dNTPs (2 μl), 5 μM forward primer (0.8 μl), 5 μM reverse primer (0.8 μl), FastPfu polymerase (0.4 μl), bovine serum albumin (0.2 μl), and 10 ng of purified template DNA, followed by adding 20 μl ddH_2_O. The thermal cycling conditions were as follows: initial denaturation at 94°C for 5 min followed by 30 cycles of 95°C for 30 s, annealing at 95°C for 30 s, and at 72°C for 45 s, and a final extension step at 72°C for 10 min. The sequencing was conducted using an Illumina MiSeq PE300 platform by Majorbio Company (Shanghai, China).

### Processing of Sequencing Data

After using the barcodes to link each sequence to its sample, the sequences were quality-trimmed based on the 50-bp window and an average quality score threshold <20. If the average quality score in the window was <20, the bases at the end of the read were truncated from the window, and reads <50 bp after quality control were filtered out using trimmomatic. Paired-end reads with ≥10-bp overlap and <5% mismatches were merged using FLASH. After removing the sequences with ambiguous bases, the raw reads were quality-filtered using QIIME software (Caporaso et al., 2010). Next, using QIIME software, the sequences were clustered into operational taxonomic units (OTUs) using a 97% sequence similarity threshold, and singleton OTUs were removed. The final OTUs were generated based on the clustering results, and taxonomic labels were assigned to the representative sequence of each OTU by the Ribosomal Database Project 16S Classifier (Cole et al., 2009). These steps were accomplished using an in-house pipeline that was based on the SILVA database.

### Statistical Analysis

We analyzed subsets of data on soil samples from two depths (0–10 and 50 cm) for each of the three regions: the E, W, and H regions. We analyzed the effects of contemporary and environmental factors on the richness and the composition of the total soil bacteria in the soil samples. All statistical tests were conducted in R v3.6.2 (R Development Core Team, 2019). To assess differences in environmental factors and α-diversity across the different sites, one-way ANOVA was performed using IBM SPSS software 19.0 (IBM Corp., Armonk, NY, United States). Box plots of the differences in α-diversity indexes (Shannon and Simpson) across samples were constructed using *ggplot* in the “VEGAN” R package. Normality was assessed using the Shapiro–Wilk test, and homogeneity of variances was assessed using Bartlett’s test, with a significance level of 0.05. Stack plots based on the phylum and family abundance matrices and simulation of grouping were constructed using the *reshape2* and *ggplot2* packages. Pairwise comparisons were made by the least significant difference test in IBM SPSS software.

A non-metric multidimensional scaling (NMDS) ordination analysis of the composition of the bacterial communities was conducted based on the Bray–Curtis dissimilarity matrix using *ggplot2* in the “VEGAN” R package. An analysis of similarities (ANOSIM) was carried out to assess the effects of the different regions and soil types on the bacterial communities. Permutational multivariate analysis of variance (PERMANOVA; 999 permutations) was performed to assess differences in community composition across regions and soil types. Redundancy analyses (RDA) were carried out to identify the associations between bacterial community structure and environmental factors; response factors were log-transformed and factors were selected for the RDA model, and the Hellinger transformation was applied to the response factors. Additionally, we used the geom-ord-ellipse function of the *yyplot* and *ggrepel* packages in “VEGAN” to add confidence ellipses.

Potential bacterial biomarkers associated with different regions and soil types were identified using the linear discriminant analysis (LDA) effect size (LEfSe) method^2^. First, non-factorial parametric Kruskal–Wallis rank-sum test was performed to identify significant differences in the abundances of the bacterial taxa. Thereafter, LEfSe was used to identify the most differently abundant taxa in various regions and soil types (Segata et al., 2011). Additionally, variation partitioning Venn diagram analysis was conducted using Venny^3^. Moreover, Spearman correlation analysis across enriched taxa and environmental factors was evaluated for significance (*P < 0.05*) using the corr.test function in the “*psych*” package in “VEGAN.” A correlation network analysis was then performed using Gephi v.0.9.1. The use of co-occurrence networks allows the interactions between functionally related bacterial communities and their responses to environmental factors to be clearly visualized. To reduce the number of rare genera in the datasets, we removed genera with a relative abundance <0.5%. Strong correlations (based on the Spearman correlation coefficient and a false-discovery-rate-corrected *P* < 0.05) were identified to construct networks in which each node signifies one genus and each edge characterizes a strong and significant correlation between two nodes. The network-level topology of the various networks was assessed in terms of the number of nodes, edges, positive interactions, and negative interactions, average degree (number of connections per node), average clustering coefficient, modularity, and average path length.

### Accession Number

Raw sequencing data regarding bacterial 16S rRNA genes were deposited in the National Center for Biotechnology Information Sequence Read Archive^3^ under BioProject accession number PRJNA631128.

## Results

### Soil Properties

Some of the environmental factors (TN and TOC) exhibited significant differences across the three regions of the Qaidam Basin of the Qinghai–Tibet Plateau, China (Table 1). However, no significant differences were found in the pH (*F* = 0.383; *P* > 0.05) or WC (*F* = 0.812; *P* > 0.05) across regions (Table 1). Regarding the subsurface soil (50 cm) and surface soil (0–10 cm), WC was higher in the subsurface soil than in the surface soil. TOC (*F* = 1.546; *P* > 0.05) was unchanged between E0 and E5 in the east region and between H0 and H5 in the high-elevation region, while it was significantly higher in W5 than W0 in the west region (Table 1). TN differed between H0 and H5 in the high-elevation region (*F* = 8.114; *P* = 0.000) but exhibited no significant differences in either the east or the west region (Table 1). As shown in Supplementary Figure 2, the two principal components accounted for 51.7% of the total environmental factor differences across individual samples. The environmental factors were distributed across the sites independently of each other, as shown in the two-dimensional plot, which indicates that the environmental factors had different values with respect to each other that changed across sites (Supplementary Figure 2).

**TABLE 1:** Environmental factors (mean ± SE) (*n* = 9) across different sites of the soil of Qaidam basin, Qinghai Tibet Plateau types sampled.

**Site**	**pH**	**WC (%)**	**TOC (g/kg)**	**TN (mg/L)**	**Elevation (m)**
E0	8.15 ± 0.28a	3.43 ± 0.38a	0.36 ± 0.07ab	33.32 ± 4.59b	3262.44 ± 66.50b
E5	8.07 ± 0.08a	4.45 ± 0.88a	0.48 ± 0.06ab	42.41 ± 3.02b	3262.44 ± 66.50b
H0	7.84 ± 0.40a	3.83 ± 0.81a	0.46 ± 0.06ab	22.23 ± 2.59a	4352.89 ± 111.44c
H5	7.95 ± 0.10a	5.02 ± 0.44a	0.45 ± 0.07ab	39.53 ± 3.17b	4352.89 ± 111.44c
W0	8.17 ± 0.17a	3.01 ± 1.00a	0.31 ± 0.05a	35.73 ± 2.81a	2825.33 ± 36.14a
W5	7.86 ± 0.19a	4.18 ± 1.03a	0.51 ± 0.08b	20.56 ± 2.23a	2825.33 ± 36.14a
*F*	0.383	0.812	1.546	8.114	81.849
*P*	>0.05	>0.05	>0.05	0.000	0.000

*Different letters indicate significant differences across sites and depth for each measurement after One-way ANOVA and Duncan comparisons. *F* and *P*-values obtained in ANOVA’s are shown at the bottom of the table.*

### Bacterial Diversity and Community Composition

There were 7,99,652 bacterial sequences, with an average length of 437.77 bp. They were clustered into 5,050 OTUs according to Good’s coverage estimator regarding the OTUs in the samples (based on 97% sequence similarity), indicating that the sequences sufficiently covered the bacterial population’s diversity in the soil samples. The rarefaction curves are presented in Supplementary Figure 3. There were major differences in the curves’ patterns across the regions. At all thresholds, the highest Shannon index was detected at high-elevation, followed by the east region and then the west region (Supplementary Figure 3).

**FIGURE 2:**
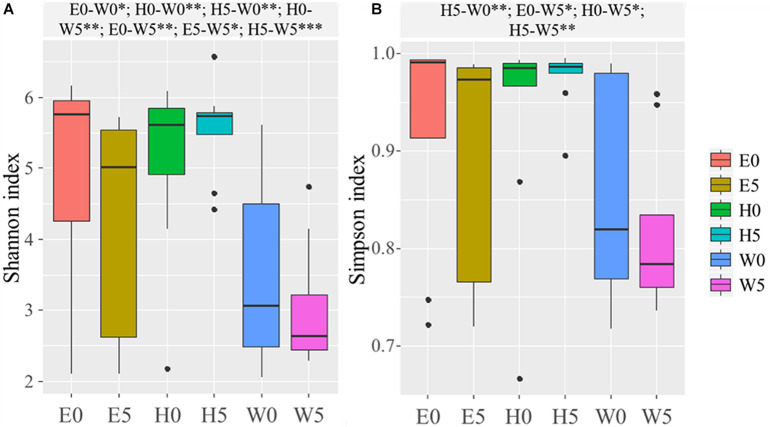
Alpha-diversity across the six sites sampled of Qaidam Basin, Qinghai–Tibet Plateau, China: **(A)** Shannon index and **(B)** Simpson index are shown in boxplots. Black bars represent the boxplot central tendency (median). While the edge of the boxes represents upper and lower quartile due to rank. Outliers are indicated with black dots. Normal distribution and variance homogeneity of the OTUs were tested using the Shapiro–Wilk test and Bartlett test functions, respectively. Pairwise comparisons made using Fisher’s least significant difference test are shown at the top of the figure. Soil samples are grouped by E – east, H – high elevation, and W – west. 0 and 5 represent depths of 0 and 50 cm, respectively. Significant codes: 0^∗∗∗^; 0.001^∗∗^; 0.01^∗^.

Based on the OTUs, the α-diversity indices (Chao, Shannon, and Simpson) were calculated (Supplementary Table 1). The Chao index (*F* = 11.634; *P* = 0.000), Shannon index (*F* = 5.493; *P* = 0.000), and Simpson index (*F* = 3.215; *P* = 0.000) were significantly lower in the W0 and W5 soil samples and remarkably higher in the H0 and H5 soil samples than all other soil samples (Supplementary Table 1). Shapiro–Wilk test and Bartlett’s test were used to assess data normality and homogeneity of variance, respectively, and Shannon index (*P* = 1.006 × 10^–5^) and Simpson index (*P* = 1.773 × 10^–7^) were then calculated for the soil samples, as shown in the boxplot in Figure 2. The Shannon and Simpson indices were significantly higher in the E0 and the W0 surface soils than the corresponding subsurface soils (E5 and W5) (Figure 2B).

**FIGURE 3:**
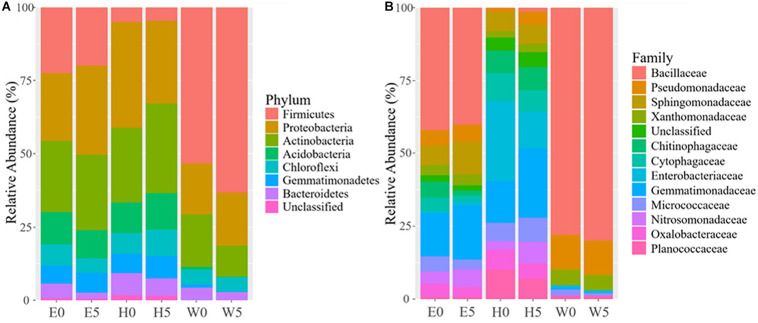
Bacterial composition across the six sites sampled of Qaidam Basin, Qinghai–Tibet Plateau, China: **(A)** relative abundance of the dominant bacterial phylum; **(B)** relative abundance of the dominant bacterial family is shown in stack plot. Soil samples are grouped by E – east, H – high elevation, and W – west. 0 and 5 represent depths of 0 and 50 cm, respectively.

**FIGURE 4:**
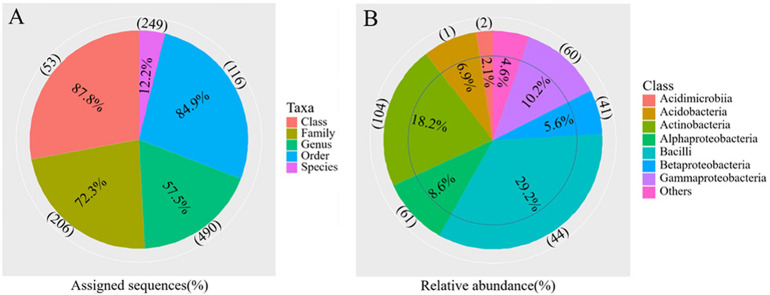
Overview of the bacterial community composition across the six sites sampled of Qaidam Basin, Qinghai–Tibet Plateau, China: **(A)** the percentage of sequences assigned (pie chart) and the number of classified taxa (in parentheses) for each taxonomic level. **(B)** The relative abundance of the eight classes and the number of classified genera (in parentheses) in each class are shown in the pie charts.

The differences in soil bacterial community structures were more apparent at higher levels of classification. At the phylum level, the bacterial communities related to the regions and soil depths were significantly different. *Firmicutes* dominated in the W0 and W5 soils, while *Proteobacteria* and *Actinobacteria* dominated in the other soils (E0, E5, H0, and H5) (Figure 3A). To get a better insight into the variations of soil bacterial community structures across the regions, we conducted functional heat map analyses of the most abundant phyla, which emphasized their relative abundances and distribution (Supplementary Figure 4). Regarding the family level, *Bacillaceae* had a significantly higher relative abundance in the W0 and W5 soils than in the other soil samples (Figure 3B). The ability to classify the sequences increased at higher taxonomic levels (Figure 4A). Regarding the class level, 53 were detected; *Bacilli* was dominant, with a relative abundance (29%) that was much higher than that of the other classes (Figure 4B).

**FIGURE 5:**
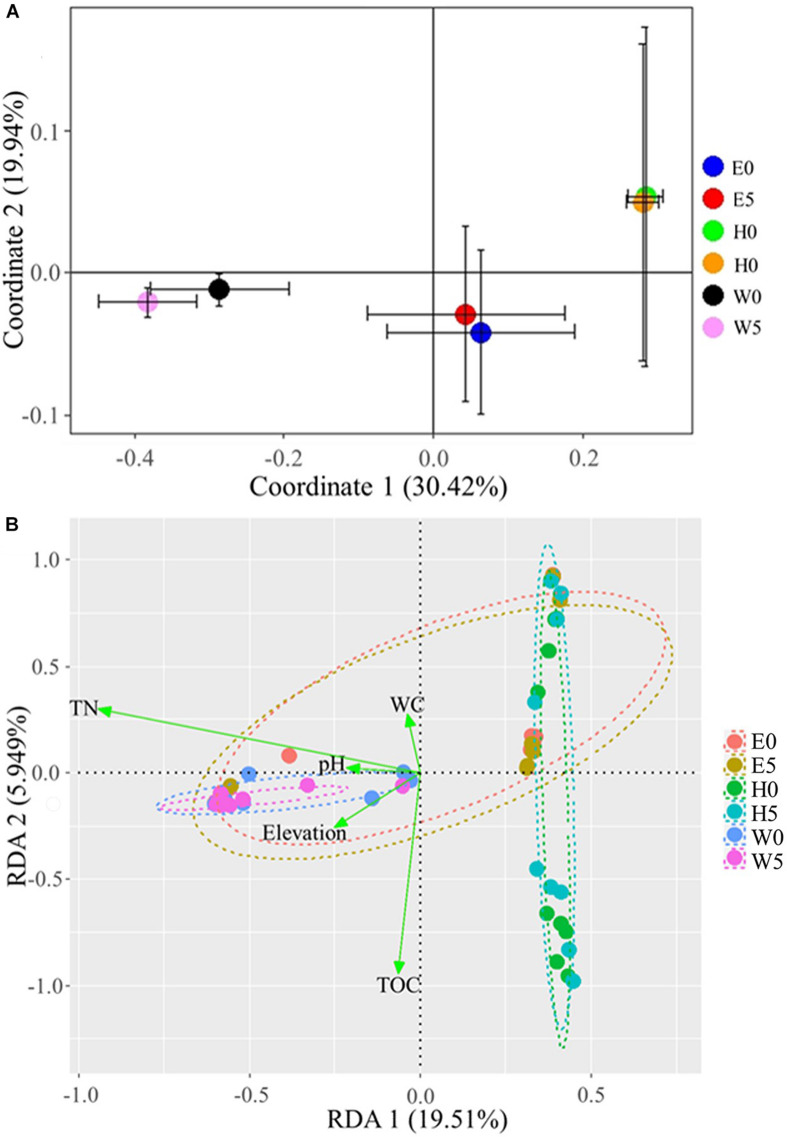
Bacterial communities across the six sites sampled of Qaidam Basin, Qinghai–Tibet Plateau, China: **(A)** principal coordinate analysis of bacterial diversity in Qaidam Basin, Qinghai–Tibet Plateau, China. Community metrics were calculated from the mean of bacterial operational taxonomic units by Bray–Curtis dissimilarity matrix. **(B)** Biplot of the redundancy analysis showing a significant relationship between environmental factors (response variables: TN, total nitrogen; WC, water content; TOC, total organic carbon) and microbial groups (explanatory variables). First and second ordination axes were plotted, representing 19.51 and 5.94% of the variability in the data set, respectively. Geom-ord-ellipse function was used; an oval contains the sample points from one site; stress = 0.1168. Soil samples are grouped by E – east, H – high elevation, and W – west.

The Bray–Curtis dissimilarities in the composition of bacterial communities across the regions were assessed in a NMDS bi-plot (Supplementary Figure 5). The bacterial communities of the surface and the subsurface soils were comparatively similar, but with some obvious differences (*R*^2^ = −0.03; *P* > 0.05) (Supplementary Figure 5). However, the three regions significantly influenced the bacterial communities (*R*^2^ = 0.36; *P* = 0.05; permutations: 999) (Supplementary Figure 5). In a more in-depth analysis, the mean bacterial OTUs across the three regions was evaluated using a PCoA (Figure 5A). Coordinate 1 explained 30.42% of the variance, while coordinate 2 explained 19.94% (Figure 5A). Clear segregation regarding the bacterial communities was detected across the regions, while surface and subsurface soils showed relatively high similarities in each region (Figure 5A).

### Effects of Environmental Factors on Bacterial Communities

To examine the possible impacts of the major environmental factors (i.e., pH, WC, TOC, TN, and elevation) on the bacterial community structures, we performed a redundancy analysis (RDA) and a Monte Carlo multivariate permutation analysis (Figure 5B). Axis 1 explained 19.51% of the variance, while axis 2 explained 5.94% (*stress* = 0.1168). Soil TN, WC, pH, and TOC were all major factors that influenced the bacterial community structures in the samples (Figure 5B). The multivariate permutation analysis showed that WC (*R*^2^ = 0.038; *P* = 0.021), TOC (*R*^2^ = 0.039; *P* = 0.027), and TN (*R*^2^ = 0.171; *P* = 0.001) significantly affected the bacterial community in each soil type (Supplementary Table 2), while the soil pH exhibited non-significant differences.

**FIGURE 6:**
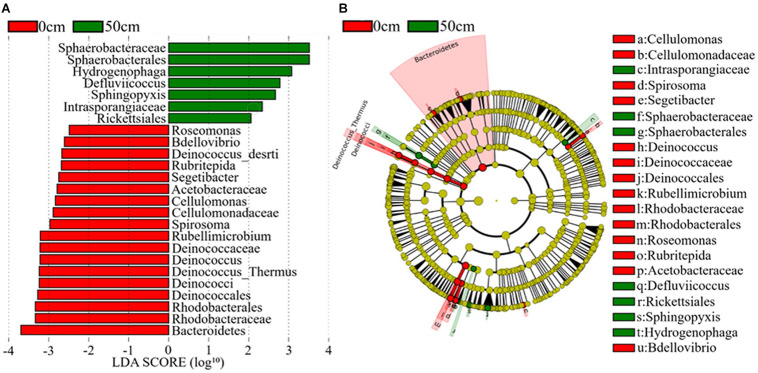
The potential biomarker was defined by LEfSe. **(A)** Histogram of the linear discriminant analysis (LDA) scores for differentially abundant features between groups (0 and 50 cm). **(B)** Cladogram for the taxonomic representation of significant differences between the surface (0 cm) and the subsurface (50 cm) groups. The threshold on the logarithmic LDA score for discriminative features was set to 3.0. Soil samples are grouped by E – east, H – high elevation, and W – west.

The bacterial taxa that most likely explain the differences between surface and subsurface soil samples from the three regions were identified based on the LEfSe (Figure 6 and Supplementary Figure 6). The results indicate that the abundances of several bacterial taxa significantly differed between the surface and the subsurface soil samples. For instance, *Bacteroidetes* and *Deinococcus–Thermus* were the main discriminant clades in the surface soil samples, while *Deinococci* was the most differentially abundant bacterial class in the subsurface soil samples (Figure 6). At the order level, *Deinococcales* and *Rhodobacterales* were significantly enriched in the surface soil samples, while *Sphaerobacterales* and *Rickettsiales* dominated in the subsurface soil samples (Figure 6). At the genus level, *Deinococcus*, *Segetibacter*, *Spirosoma*, *Cellulomonas*, *Bdellovibrio*, *Rubellimicrobium*, and *Rubritepida* exhibited relatively higher abundances in the surface soil samples, while *Hydrogenophaga* and *Sphingopyxis* exhibited relatively higher abundances in the subsurface soil samples (LDA > 3, *P* < 0.05) (Figure 6). Supplementary Figure 6 shows the cladograms highlighting the potential biomarkers of different soil sample groups. The abundances of the families *Deinococcaceae*, *Sphaerobacteraceae*, *Intrasporangiaceae*, *Cellulomonadaceae*, and *Acetobacteraceae* were significantly different among the E0 soil samples, but not among the E5 soil samples (Supplementary Figure 6A). Moreover, *Micromonosporaceae*, *Coxiellaceae*, and *Rhodospirillaceae* were the dominant families in the H5 soil sample, while *Comamonadaceae* was dominant in the H0 soil samples (Supplementary Figure 6B). Furthermore, *Nannocystaceae* was a highly dominant family in the W0 soil samples, but not in the W5 (LDA score > 3, *P* < 0.05) (Supplementary Figure 6C).

### Co-occurrence Interactions Between Bacterial Communities and Environmental Factors

As shown in Supplementary Figure 7, *Firmicutes* was significantly positively correlated with TN but negatively correlated with elevation. Additionally, *Proteobacteria*, *Actinobacteria*, *Acidobacteria*, and *Gemmatimonadetes* were negatively correlated with TN but positively correlated with elevation. TOC was positively correlated with *Proteobacteria* but negatively correlated with *Acidobacteria* and *Gemmatimonadetes*. WC and pH were not significantly correlated (Supplementary Figure 7).

**FIGURE 7:**
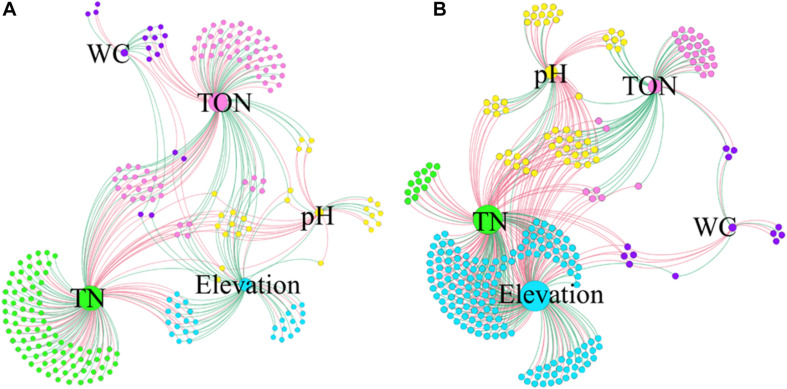
Co-occurrence networks of genera associated with environmental factors. The samples from 0 cm **(A)** to 50 cm **(B)** were separately analyzed at the genus level. Each node infers a significant correlation genus; each edge means correlated genera with Spearman’s correlation at 0.05 significance level. Only those significantly related (*P* < 0.05) to environmental factors were the concern of this study. The red lines represent the negative correlation; the green lines represent the positive correlation. The size of each node is proportional to the number of connections, that is, the degree (20–100). Each node is labeled at the genus level, and the expansion scale factor is (1.2).

The co-occurrence interactions at the genus level were visualized in a network analysis, showing significant variations with environmental factors. The network analysis is presented in Figure 7 and Supplementary Figure 8, and the details are shown in Supplementary Table 3. Overall, the surface soil samples had the lowest number of edges (335), with the lowest average degree (2.815). Additionally, the surface soil samples exhibited 49.55% positive and 50.45% negative correlations, and the highest modularity (0.450) (Figure 7A and Supplementary Table 3). On the other hand, the subsurface soil samples had the highest number of edges (483), indicating a high amount of pairwise co-variation of genera, with a higher average degree (3.687). Additionally, the subsurface soil samples exhibited 36.44% positive and 63.56% negative correlations and the lowest modularity (0.289) (Figure 7B and Supplementary Table 3). Furthermore, across soil types and regions, network interactions were analyzed at the genus level to identify correlations with environmental factors, with positive and negative correlations being assessed (Supplementary Figure 8 and Supplementary Table 3). The E0 bacterial network had the highest number of edges (123), and the W5 network had the lowest (22). The H5 network had the highest average degree (2.388), 54.99% positive and 47.01% negative interactions, whereas the W5 network had the lowest average degree (1.760), with 50% positive and 50% negative genera interactions (Supplementary Figure 8 and Supplementary Table 3). These results showed that both the soil types and the regions had significant impacts on the bacterial community interactions.

## Discussion

This study presents a complete assessment of soil ecosystem bacterial diversity in surface and subsurface niches at the lower temperature limit for life and provides insights into ecosystem complexity under extreme stresses. The highly specialized bacterial communities in these niches face severe environmental challenges/factors; as a result, these bacterial communities adapt several protective strategies to cope with this hostile environment (Sajjad et al., 2020a). Several reports over the last decade have revealed that some bacterial taxa are distributed in restricted ranges of environmental factors (Van Horn et al., 2013; Griffiths et al., 2016; Wang et al., 2017). As the present global climate change effects are most pronounced in desert regions and as there could be increases in localized cooling in the Qaidam Basin (Xing et al., 2019), it is timely to explore this endangered environment.

### Bacterial Diversity in the Qaidam Basin

The desert ecosystem of the Qaidam Basin is a rich reservoir for bacteria. The surface soil samples had higher α-diversity indices (Chao, Shannon, and Simpson) (Supplementary Table 1 and Figure 2), the highest modularity index in the co-occurrence network, and the largest number of biomarker taxa. The level of diversity in this study indicates a more complex ecosystem in the Qaidam Basin than previously reported by Xing et al. (2019). The present data showed that the view based on soil analyses indicate that bacterial phyla such as *Firmicutes*, *Proteobacteria*, *Actinobacteria*, *Acidobacteria*, *Chloroflexi*, *Gemmatimonadetes*, and *Bacteroidetes* are dominant in both surface and subsurface soil samples across the region (Figure 3). This finding is in accordance with previous studies of soil samples (Constancias et al., 2015; Xing et al., 2019). The widespread phyla such as *Actinobacteria* and *Proteobacteria* that are involved in soil development are dispersed by aerosolized soil dust and can colonize new environments (Hill et al., 2011; Zhang et al., 2012; Barberan et al., 2014). Similarly, such adaptations have been reported regarding the Qatari desert, the Namib Desert (Valverde et al., 2015; Abdul Majid et al., 2016), the Negev Desert (Saul-Tcherkas and Steinberger, 2011), and the hyper-arid Atacama Desert (Connon et al., 2007). Additionally, a previous study reported less diverse distributions of rare phyla, which might be connected to their restricted migration abilities (Galand et al., 2009). Furthermore, we detected *Chloroflexi* and *Gemmatimonadetes* across the region, which have been observed in previous studies (Chu et al., 2010; Nunes, da Rocha et al., 2015). However, another study on arid and hyper-arid deserts did not find either *Chloroflexi* or *Gemmatimonadetes* (Angel and Conrad, 2013). The different findings between this study and other studies (Steven et al., 2012; Angel and Conrad, 2013) might be linked to the use of different sequencing methods as well as differences in geographical location. Furthermore, the family *Bacillaceae* was the most abundant taxon in the Qaidam Basin in our study, and this was also the case in the desert soil crust (Nunes, da Rocha et al., 2015). In our study, the consistency regarding phyla and families at different soil depths within the same regions and the differences between the three regions indicate that regional factors broadly dictate the basic bacterial taxonomic compositions.

### Regional Effects on Bacterial Diversity

There was a clear picture of large differences in soil bacterial community structures across the regions and smaller differences between different soil depths (Figure 5 and Supplementary Figure 5). According to Martiny et al. (2011) and Bahram et al. (2016), the bacterial community structure on a global scale depends on precipitation and temperature. However, the present study was on a regional scale. In our study, the soil biota was dominated by desiccation- and radiation-tolerant taxa such as *Deinococcus* and *Bacteroidetes*, which have been observed in several other soil environments (Aislabie et al., 2006; Niederberger et al., 2008; Peng et al., 2009a,b; Pointing et al., 2009). However, the bacterial community in the Qaidam Basin was diverse compared to the results of previous studies. In contrast to our study, Takahashi et al. (1996), Okoro et al. (2009), and Ding et al. (2012) reported highly diverse bacterial communities in desert habitats. In addition, *Geodermatophilaceae* was only found in the E0 samples (surface soil in the east region) in our study, but it has been reported in the Heihe River Basin of Northwest China (Zhang et al., 2019) and was found to be dominant in both the Tengger and the Badain Jaran deserts in China (Sun et al., 2018). Furthermore, *Hydrogenophaga* was detected in our study, but not in other studies of deserts. These observations regarding the potentially unique bacterial communities indicate the existence of multiple ecological roles that may reflect the trends related to climate change in the Qaidam Basin.

### Effects of Environmental Factors on Bacterial Community Structure

Our multivariate permutation analysis indicated that TN, TOC, and WC were the major driving factors shaping the bacterial community structures (Supplementary Table 2). Several studies have found that the soil bacterial communities are strongly shaped by soil properties, such as pH, TOC, Mg^2+^, and WC (Fierer et al., 2003; Chu et al., 2010; Xing et al., 2019). In particular, soil pH has been previously identified as a key factor influencing bacterial community structure (Fierer and Jackson, 2006; Lauber et al., 2009). However, for soils with a very high pH and low precipitation, the driving factors of bacterial community structure are more difficult to determine (Fierer and Jackson, 2006; Lauber et al., 2009; Rousk et al., 2010). In this study, the pH was high, and it was not significantly associated with the bacterial populations. The pH signifies the presence of metals, and it modifies the enzymatic activity of soil bacteria and affects the obtainability of nutrients (Rousk et al., 2010; Ren et al., 2018). Therefore, whether pH-dependent soil factors or the pH itself shapes bacterial communities cannot be easily determined (Rousk et al., 2010). Limited attention was paid to the influence of TN and TOC in studies. In our study, soil TN and TOC were significantly correlated with the relative abundance of bacterial phyla, which is in line with several previous studies (Ganzert et al., 2011; Stomeo et al., 2012; Wang et al., 2017). However, TN and TOC did not exhibit similar patterns between the surface and the subsurface soil samples across the three regions (Table 1), and they might be the driving factors underlying unique bacterial community structures. The abundance of *Firmicutes* was significantly correlated with TN because it can produce endospores and consequently cope with extreme conditions. Additionally, the abundance of *Proteobacteria* was significantly correlated with TOC and TN (Supplementary Figure 7). This is a very exciting finding because many halophilic bacteria are members of this phylum (Oren, 2002). Our study provides evidence that a great number of halophilic and halo-tolerant bacteria that are potential industrial assets are present in the Qaidam Basin; hence, further research is required.

The co-occurrence network analysis showed that TN and TOC exhibited the maximum number of associations with bacterial genera in the surface soil, while the high-elevation exhibited the maximum number of positive associations with bacterial genera in the subsurface soil (Figure 7 and Supplementary Figure 8). Interestingly, the subsurface soil was highly negatively correlated in the co-occurrence network analysis. While TN exhibited the maximum number of associations with bacterial genera in both the surface and the subsurface soil samples in the east region, TOC exhibited the maximum number of associations with bacterial genera in both the surface and the subsurface soil samples at high elevation. Dominant positive correlations indicate that most bacterial taxa may act synergistically or share similar ecological niches regarding the environmental factors. Our results indicate the presence of potentially valuable bacterial community members in the desert regions of the Qaidam Basin, including rare community members that might have significant ecological roles. Previous research compared the relationships between bacterial communities and both spatial distance and environmental factors (Fierer and Jackson, 2006; Martiny et al., 2011). Our results indicate that environmental factors play a crucial role in shaping the soil bacterial community structure in the Qaidam Basin. These results support the findings on bacterial communities in the Western Tibetan Plateau and other regions (Griffiths et al., 2011; Chu et al., 2016; Xing et al., 2019).

## Conclusion

In conclusion, this study provides information on the regional patterns of bacterial diversity in the extremely harsh environment of the Qaidam Basin of the Qinghai–Tibet Plateau. In addition to precipitation and temperature, which are considered to be driving factors in global-scale studies, other factors such as TN, TOC, WC, and pH might be the main driving factors of bacterial community structure in regional-scale studies. Our study provides information on the unique patterns of environmental factors both in surface and subsurface soils that acted as driving factors for bacterial community structure across the regions of the Qaidam Basin. The main driving factors that significantly influenced the bacterial diversity and community composition in the cold desert soil samples were TN and TOC. Moreover, phyla such as *Firmicutes*, *Proteobacteria*, and *Actinobacteria* were dominant across the regions of the Qaidam Basin. The Qaidam Basin is an interesting ecosystem that needs to be further explored in terms of its unique bacterial community and the role of microbial diversity in global warming.

## Data Availability Statement

Raw sequencing data of regarding the bacterial 16S rRNA genes were deposited in the National Center for Biotechnology Information (NCBI) Sequence Read Archive (https://pubmed.ncbi.nlm.nih.gov/) under BioProject accession number PRJNA631128.

## Author Contributions

WZ and AB performed the experiments and prepared the manuscript. BZ helped prepare some experiments. GZ and BZ contributed in sampling from Qaidam Basin. AB performed the statistical analysis. GZ, XW, TC, and GL helped correct the manuscript. All authors contributed to the article and approved the submitted version.

## Conflict of Interest

The authors declare that the research was conducted in the absence of any commercial or financial relationships that could be construed as a potential conflict of interest.

## Footnotes

1 http://data.cma.cn

2 http://huttenhower.sph.harvard.edu/lefse/

3 https://bioinfogp.cnb.csic.es/tools/venny/index.html
