# Supplementary material for: Diverse Bacterial Communities From Qaidam Basin of the Qinghai–Tibet Plateau: Insights Into Variations in Bacterial Diversity Across Different Regions
Source: Front Microbiol. 2020 Sep 18;11:554105. doi: 10.3389/fmicb.2020.554105 (PMC7530167; doi:10.3389/fmicb.2020.554105)
Supplement: Supplementary file 1 [file Data_Sheet_1.docx]

**Supplementary Data**

**Supplementary Table 1.** Alpha-diversity (mean ± SE) (n = 9) across different sites sampled of Qaidam Basin, Qinghai Tibet Plateau. Different letters indicate significant differences across locations for each measurement after ANOVA and Duncan comparisons. *F* and *P*-values obtained in ANOVA’s are shown at the bottom of the table.

| **Diversity index** | | | | | |
| --- | --- | --- | --- | --- | --- |
| **Site** | **Chao** | **Fisher** | **Evenness** | **Shannon** | **Simpson** |
| E0 | 1075±165.5b | 196.4±35.8a | 0.22±0.04c | 4.9±0.53c | 0.93±0.04bc |
| E5 | 991±151.7ab | 165.3±30.8ab | 0.13±0.03abc | 4.3±0.51bc | 0.90±0.04abc |
| H0 | 1633±112.4c | 291.9±24.9c | 0.17±0.03bc | 5.1±0.41c | 0.94±0.04bc |
| H5 | 1693±179.6c | 321.0±41.4c | 0.20±0.03bc | 5.6±0.22c | 0.98±0.01c |
| W0 | 641±105.0a | 95.2±19.9a | 0.11±0.04ab | 3.5±0.46ab | 0.85±0.04ab |
| W5 | 612±82.6a | 89.3±14.8a | 0.05±0.01a | 3.0±0.29ab | 0.82±0.03ab |
| *F* | 11.634 | 10.941 | 4.026 | 5.493 | 3.215 |
| *P* | 0.000 | 0.000 | 0.004 | 0.000 | 0.014 |

**Supplementary Table 2.** Results of perMANOVA analysis of the Bray-Curtis dissimilarities for bacterial OTU community structure in pH, WC, TOC, TN, and Elevation. Df = degrees of freedom; SS = sum of squares; MS = mean sum of squares; Pseudo-F = F value by permutation. *P*-values are based on 9999 permutations.

|  | **Df** | **SS** | **MS.** | ***F*** | ***R^2^*** | ***P*** |
| --- | --- | --- | --- | --- | --- | --- |
| pH | 1 | 0.5763 | 0.5763 | 2.0077 | 0.02930 | 0.054· |
| WC | 1 | 0.7400 | 0.7400 | 2.5780 | 0.03763 | 0.021 * |
| TOC | 1 | 0.7595 | 0.7595 | 2.6458 | 0.03862 | 0.027 * |
| TN | 1 | 3.3685 | 3.3685 | 11.7346 | 0.17127 | 0.001 *** |
| Elevation | 1 | 0.4447 | 0.4447 | 1.5493 | 0.02261 | 0.141 |

Significant codes: 0 ‘***’; 0.001‘**’; 0.01 ‘*’; 0.05 ‘.’

| **Network attributes** | **0cm** | **50cm** | **E0** | **E5** | **H0** | **H5** | **W0** | **W5** |
| --- | --- | --- | --- | --- | --- | --- | --- | --- |
| Nodes | 238 | 262 | 104 | 102 | 103 | 98 | 42 | 25 |
| Edges | 335 | 483 | 123 | 98 | 109 | 117 | 47 | 22 |
| Int. Positives | 49.55% | 36.44% | 45.53% | 56.12% | 55.96% | 54.99% | 78.72% | 50% |
| Int. Negatives | 50.45% | 63.56% | 54.47% | 43.28% | 44.04% | 47.01% | 21.28% | 50% |
| Average Degree | 2.815 | 3.687 | 2.365 | 1.922 | 2.117 | 2.388 | 2.238 | 1.760 |
| Modularity | 0.450 | 0.289 | 0.430 | 0.410 | 0.431 | 0.409 | 0.445 | 0.677 |
| Avg. Clustering Coef. | 0.000 | 0.000 | 0.000 | 0.000 | 0.000 | 0.000 | 0.000 | 0.000 |

**Supplementary Table 3.** Topological parameters of network analysis of each site and group.

**
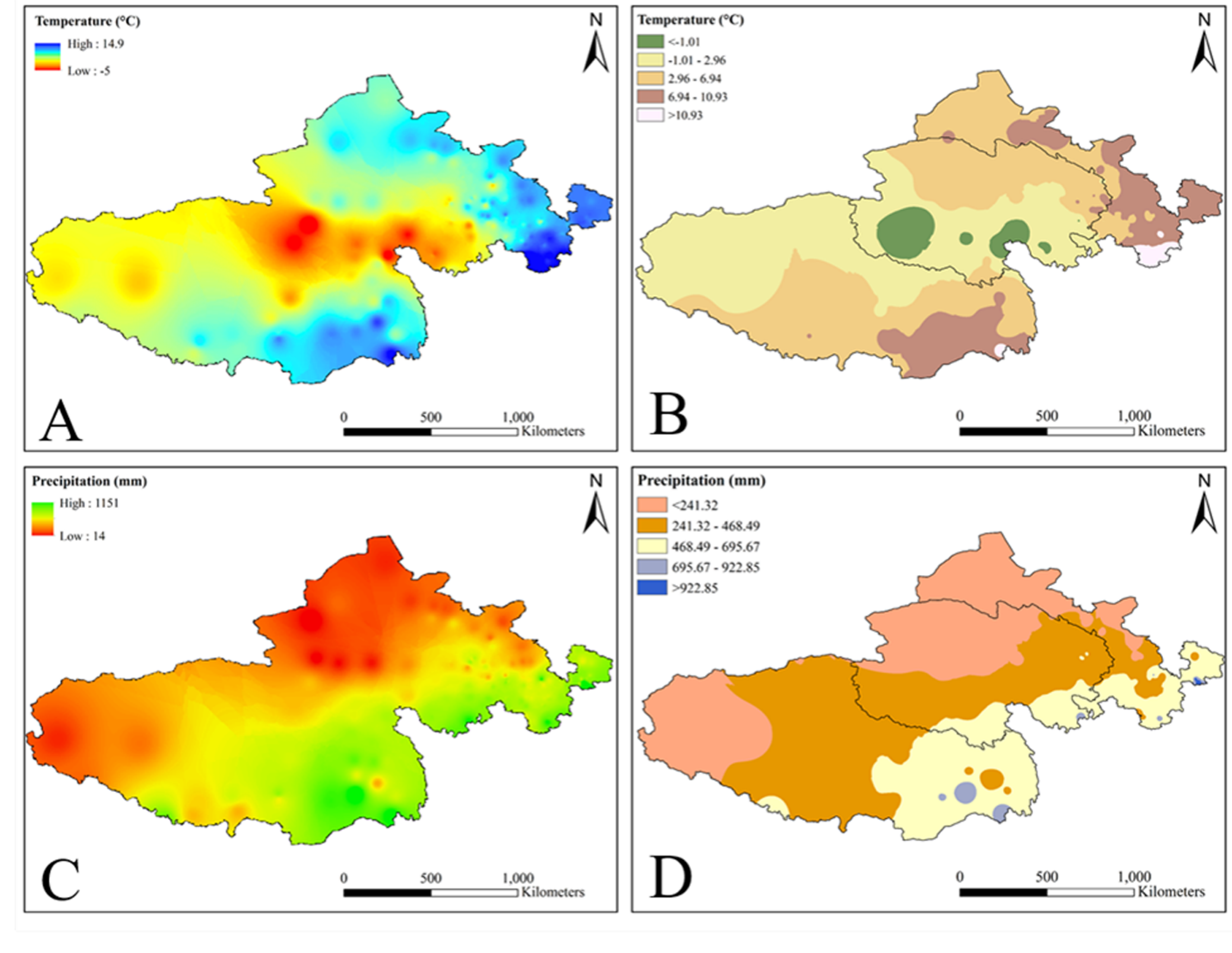
** **Supplementary Figure 1.** **(A)** Temperature data obtained from ordinary Inverse distance weighting (IDW) interpolation based on the 30-year average annual records (1981 to 2010). **(B)** Map where similar sites have been recognized giving to temperature intervals indicated by different colors. **(C)** Precipitation map obtained from ordinary Inverse distance weighting (IDW) interpolation based on the 30-year average annual records (1981 to 2010). **(D)** Map where similar sites have been recognized giving to precipitation intervals indicated by different colors.


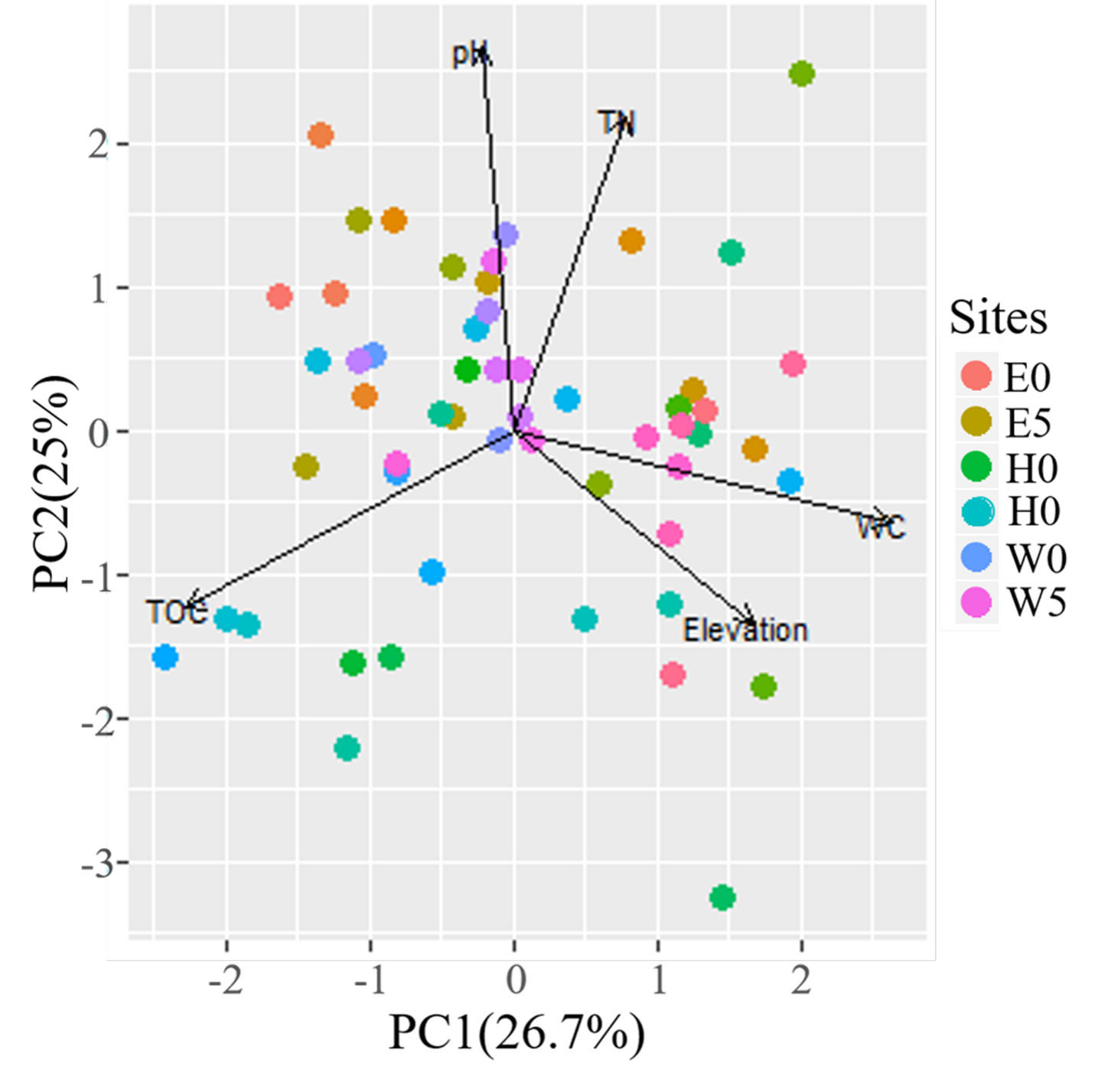


**Supplementary Figure 2.** Principle component analysis (PCA) of determined soil properties of the Qaidam Basin, Qinghai Tibet Plateau, China (including soil TOC = total organic carbon; TN = total nitrogen; soil pH; WC = water content, Elevation). Soil samples are grouped by E-East, H-high Elevation, and W-West. 0 and 5 represent depths of 0 cm and 50 cm respectively.


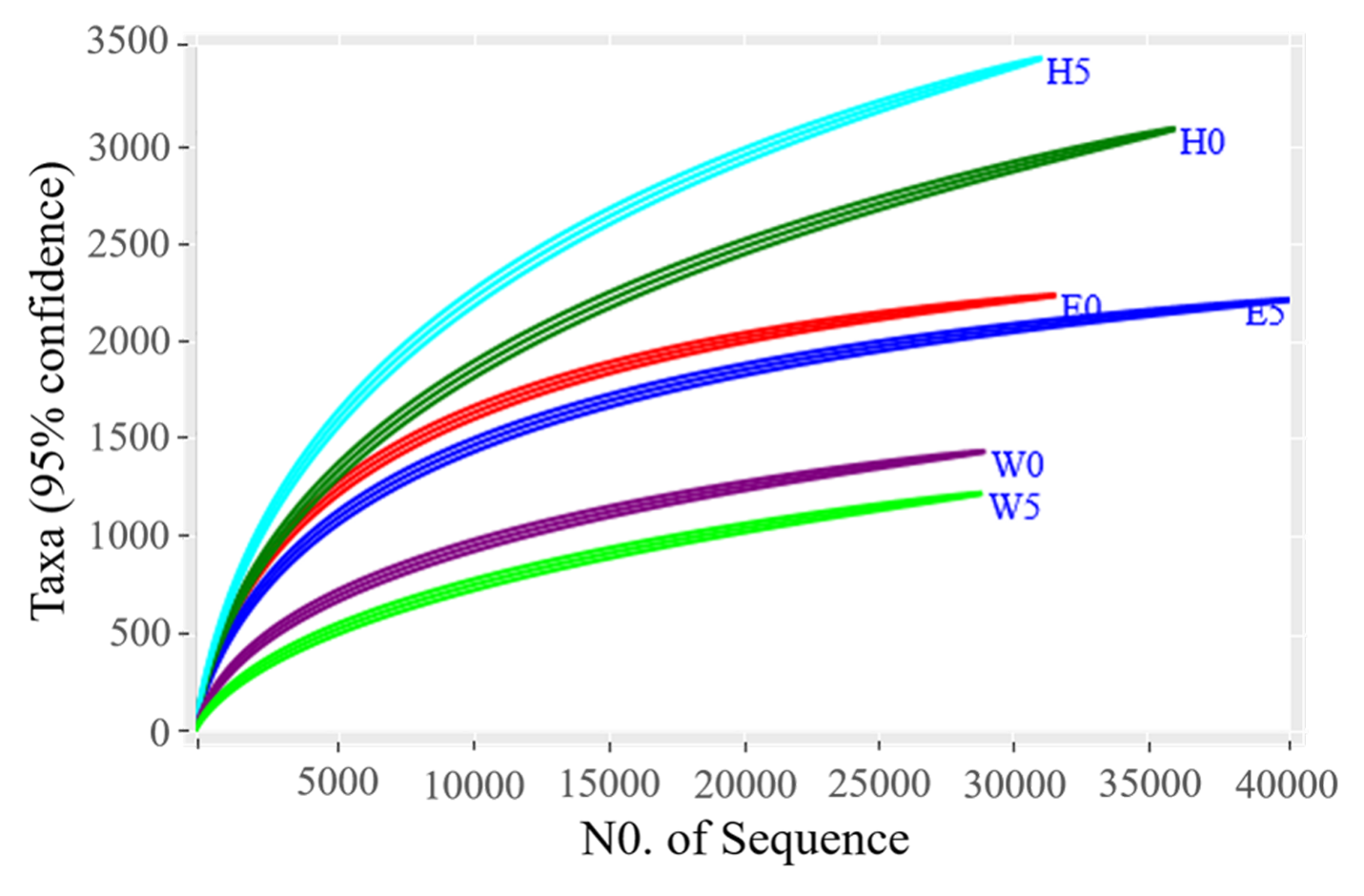


**Supplementary Figure 3.** Rarefaction curves of all sequences of soil samples.


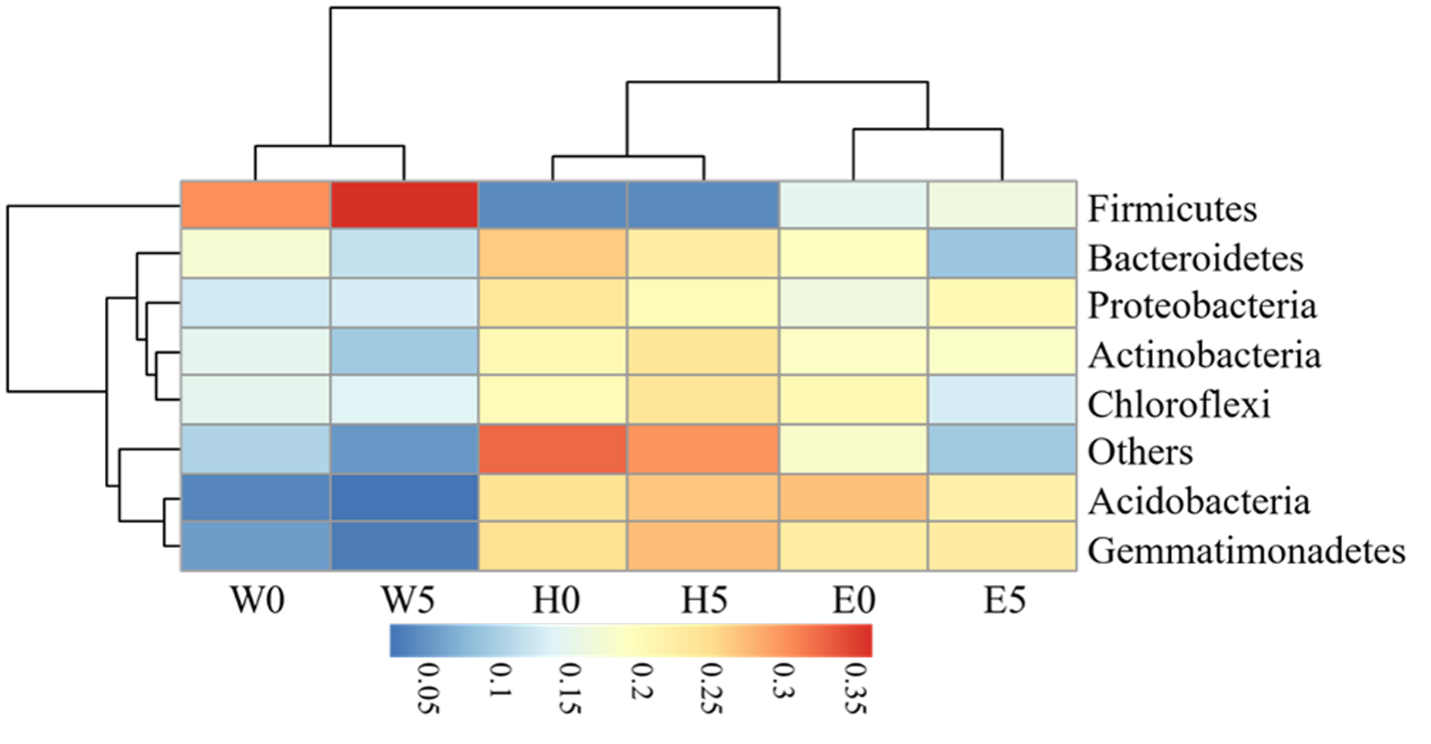


**Supplementary Figure 4.** Heat map of top 7 phyla in all samples. The color intensity (log scale) in each panel shows the percentage of phyla in a sample, referring to the color key at the right. China. The top and the left graph show the cluster results of the bacterial community at the level of phylum by the Bray–Curtis method.


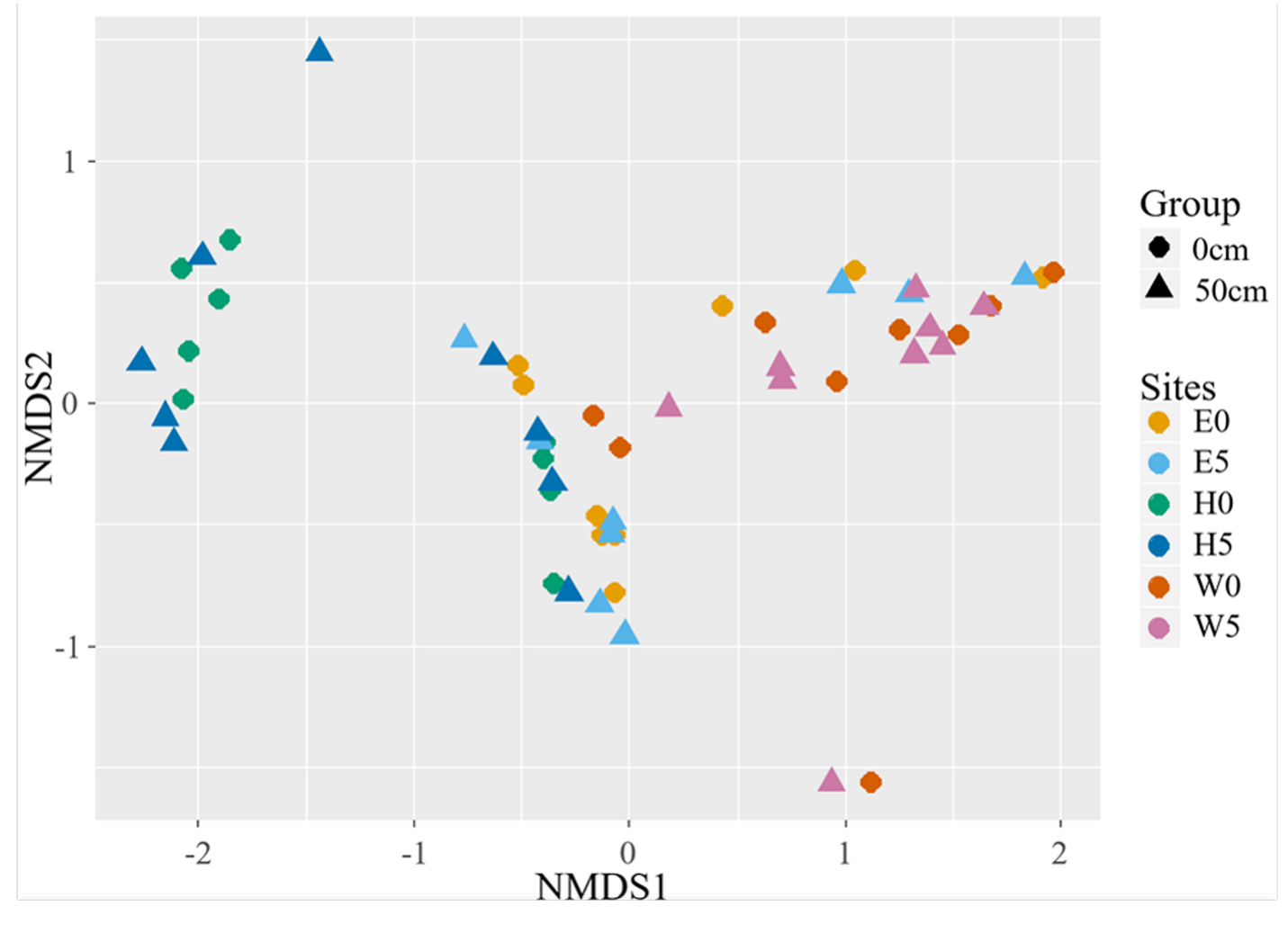


**Supplementary Figure 5.** Non-metric multidimensional scaling (nMDS) biplot of the Bray–Curtis-based dissimilarity matrix of bacterial community’s structure. Soil samples are divided into group (circle = 0 cm; triangle = 50 cm). ANOSIM R metric, a number indicating the degree of differences between the group (*R^2^* = -0.03; *P* > 0.05) and across the sites sample (*R^2^* = 0.36; *P* = 0.000), for comparisons of bacterial communities.


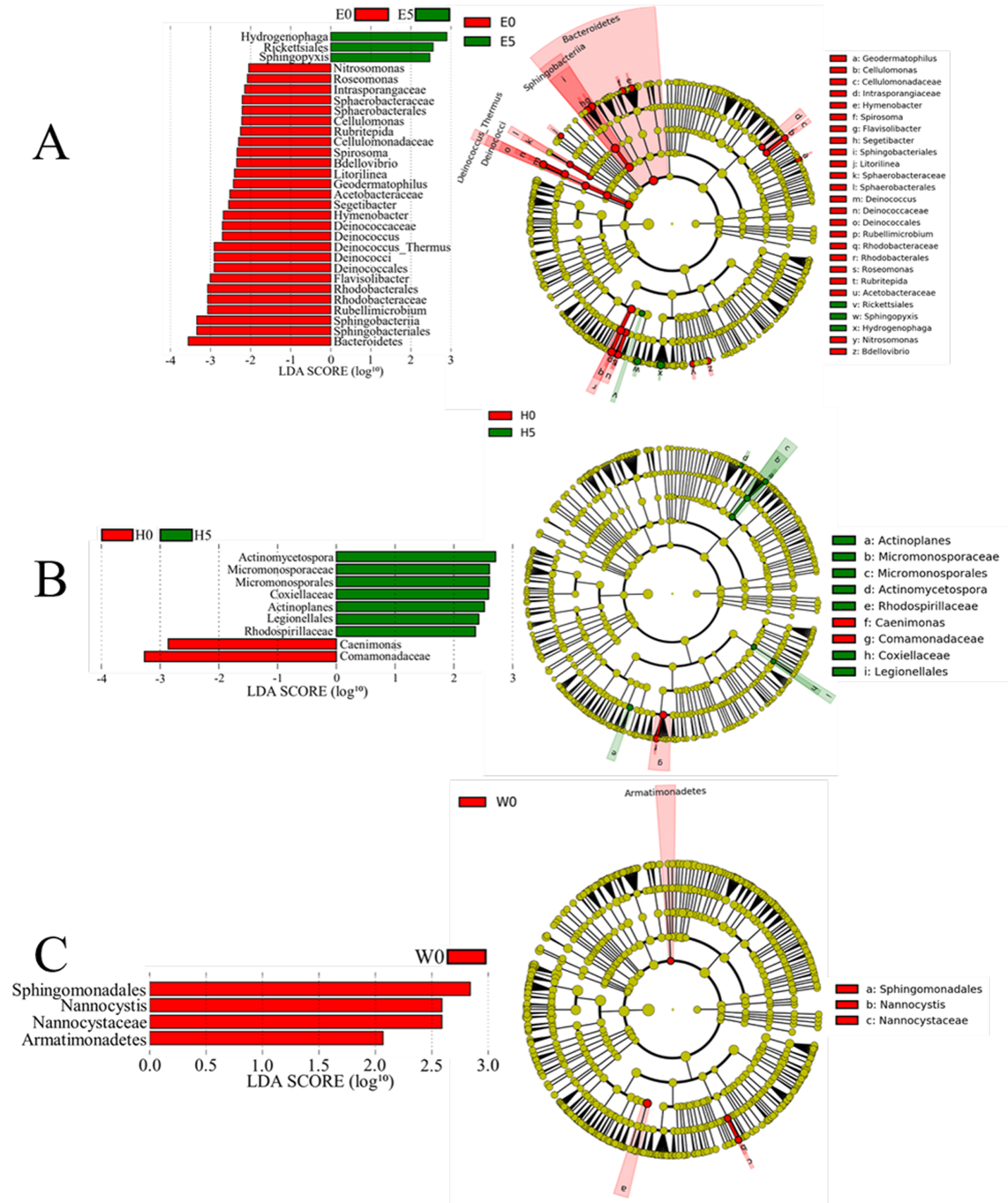


**Supplementary Figure 6.** The potential biomarkers were defined by LEfSe. **(A, B, C)** Histogram of the LDA scores and Cladogram for the taxonomic representation of significant differences between the surface (0cm) and subsurface (50cm) groups in each site. Soil samples are group by E-East, H-high Elevation, and W-West. LDA score for discriminative features was set to 3.0.


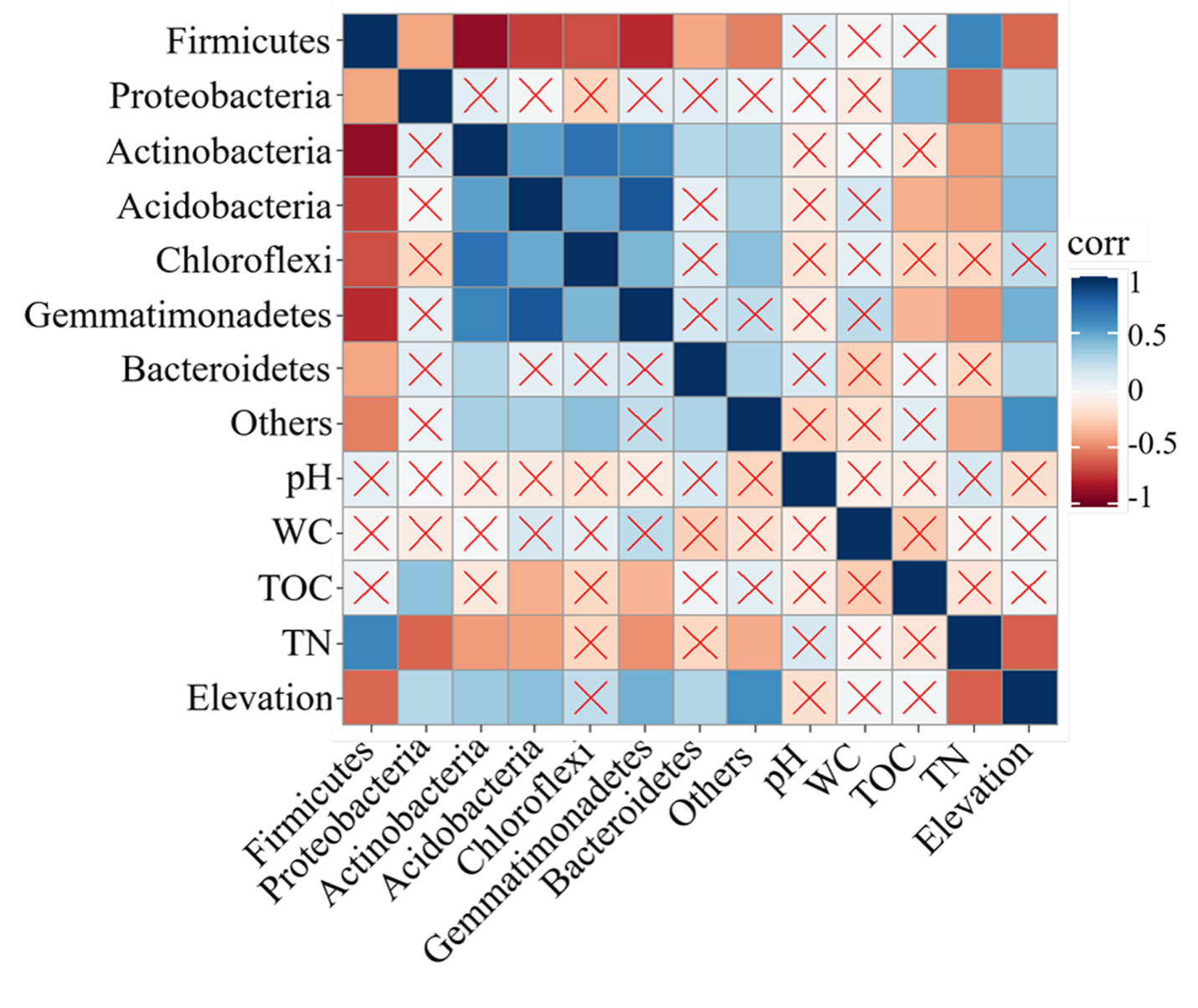


**Supplementary Figure 7.** Pearson-correlation matrix for soil properties associated with dominant bacterial phyla. The scale on the right indicates the correlation strength.


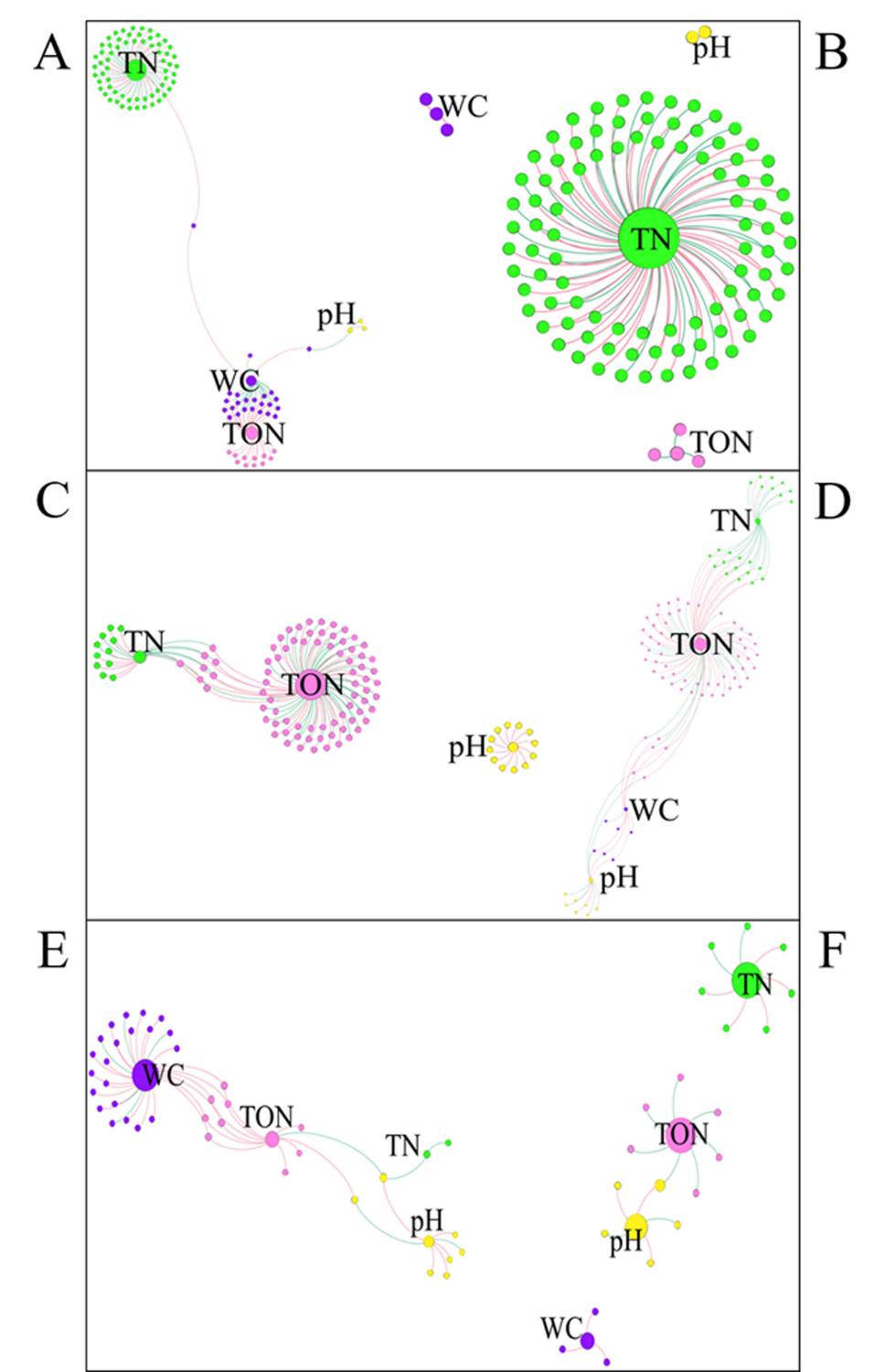


**Supplementary Figure 8.** Co-occurrence networks of genera associated with environmental factors. The samples from E0 **(A)**, E5 **(B)**, H0 **(C)**, H5 **(D)**, W0 **(E)**, and W5 **(F)** were separately analysed at the genus level. Each node infer a significant correlation genus, each edge means correlated genera with Spearman’s correlation at the 0.05 significance level. Only the significantly related (*P* < 0.05) to environmental factors were concerned in this study. The red lines represent the negative correlation, the green lines present the positive correlation. The size of each node is proportional to the number of connections, that is, the degree.
